# Glucagon and exenatide improve contractile recovery following ischaemia/reperfusion in the isolated perfused rat heart

**DOI:** 10.14814/phy2.15597

**Published:** 2023-03-22

**Authors:** Ross T. Lindsay, Philip Ambery, Lutz Jermutus, Andrew J. Murray

**Affiliations:** ^1^ Department of Physiology, Development and Neuroscience University of Cambridge Cambridge UK; ^2^ Research and Early Development, Cardiovascular, Renal and Metabolism, BioPharmaceuticals R&D, AstraZeneca Cambridge UK; ^3^ Late‐stage Development, Cardiovascular, Renal and Metabolism, BioPharmaceuticals R&D, AstraZeneca Gothenburg Sweden

**Keywords:** cardiac ischaemia/reperfusion, contractile recovery, GLP‐1 receptor agonist, glucagon

## Abstract

The inotropic effects of glucagon have been recognized for many years, but it has remained unclear whether glucagon signaling is beneficial to cardiac function. We evaluated the effects of glucagon alone and in combination with the glucagon‐like peptide 1 (GLP‐1) receptor agonist exenatide in the isolated perfused rat heart. The isolated perfused rat heart was used to investigate the initial inotropic and chronotropic effects of glucagon and exenatide during aerobic perfusion, and recovery of contractile function following ischaemia/reperfusion. Glucagon, but not exenatide, elicited an acute chronotropic and inotropic response during aerobic perfusion of the rat heart. Compared with control, glucagon improved recovery of left ventricular developed pressure (LVDP) by 33% (*p* < 0.05) and rate‐pressure product (RPP) by 66% (*p* < 0.001) following ischaemia/reperfusion and amplified the mild recovery enhancement elicited by exenatide in a dose‐dependent manner. Glucagon shows inotropic properties in the isolated perfused rat heart and improves contractile recovery following ischaemia/reperfusion, both alone and when co‐administered with a GLP‐1 receptor agonist. Glucagon and exenatide, a GLP‐1 receptor agonist, combine to stimulate greater recovery of postischaemic contractile function in the Langendorff heart. Glucagon was inotropic and chronotropic, yet this initial effect decreased over time and did not account for the increased contractility observed postischaemia/reperfusion.

## Graphical abstract

Glucagon and exenatide, a glucagon‐like peptide 1 (GLP‐1) receptor agonist, combine to stimulate greater recovery of post‐ischaemic contractile function in the Langendorff heart. Glucagon was inotropic and chronotropic, yet this initial effect decreased over time and did not account for the increased contractility observed post ischaemia/reperfusion.

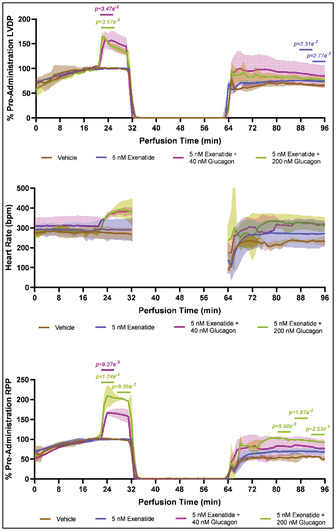

## 1 INTRODUCTION

In addition to its metabolic effects, glucagon has been shown to be a cardio‐stimulant, which increases heart rate and myocardial contractility (chronotropic and inotropic effects) (Ceriello et al., 2016). Successful administration of glucagon has been reported in a range of cardiovascular disorders, including heart failure and cardiogenic shock (Lvoff & Wilcken, 1972; Parmley et al., 1968; White, 1999). Lvoff and Wilcken noted the beneficial effects of administering glucagon to patients with severe ischaemic heart disease (Lvoff & Wilcken, 1972). Glucagon has also been a first‐choice treatment for β‐blocker intoxication (Rotella et al., 2020; White, 1999), although this clinical efficacy has not been assessed in a controlled clinical trial.

Glucagon receptor agonism, as a key component of unimolecular incretin poly‐agonists such as glucagon‐like peptide 1 (GLP‐1)/glucagon “dual” or GLP‐1/glucose‐dependent insulinotropic polypeptide (GIP)/glucagon “triple” receptor agonists, is now being investigated in the treatment of type 2 diabetes mellitus, obesity, diabetic kidney disease, and nonalcoholic steatohepatitis (Ambery et al., 2018; Bossart et al., 2022; Jia et al., 2021; Parker et al., 2020, 2022). Glucagon receptor agonism leads to an improvement in hepatic mitochondrial function, and long‐term dual glucagon and GLP‐1 receptor agonism have positive effects on glucose control, weight management, lipid profile, and liver function (Ambery et al., 2018; Boland et al., 2020; Nahra et al., 2021).

The heart has been shown to be one of the multiple tissues in the body, which express the glucagon receptor (Charron & Vuguin, 2015), albeit at a lower level than the liver or kidney. This, coupled with the inotropic and metabolic effects described, suggests that glucagon could protect the heart against ischaemia/reperfusion injury, yet studies addressing this to date have painted a mixed picture. Some studies have shown improved vasodilation postischaemia in the rat heart, and cardiac benefits in humans (Lvoff & Wilcken, 1972; Rosic et al., 2010; White, 1999). Others, using the mouse heart, suggested instead that glucagon antagonism postreperfusion may improve remodeling and ejection fraction, and that glucagon agonism may impair contractile recovery postreperfusion (Ali et al., 2015; Karwi et al., 2019). Preischaemic inotropism, seen in rats, dogs, cats, guinea pigs, and humans (Farah & Tuttle, 1960; Rodgers et al., 1981; Rotella et al., 2020; White, 1999), was not observed in these mouse studies (Ali et al., 2015), suggesting that inter‐species variation may explain the discrepancies between studies.

GLP‐1 agonism has been shown to protect the heart against ischaemia/reperfusion (Aravindhan et al., 2015; Nikolaidis et al., 2004), and the GLP‐1 receptor is present in all four chambers of the heart (Baggio et al., 2018). However, GLP‐1 agonism has the effect of enhancing glycolysis while decreasing fat oxidation (Aravindhan et al., 2015), which conflicts with the healthy heart's predominant reliance on fatty acid oxidation (Neely et al., 1967). GLP‐1 agonism also lowered cAMP and increased lactate production in the left ventricle, which may potentially temper any beneficial effects (Aravindhan et al., 2015). In the liver, the combination of GLP‐1 agonism with glucagon agonism led to enhanced mitochondrial metabolism and fatty acid oxidation and the myocardial effects of combined agonism would therefore be of interest.

The effect of glucagon on the ischaemic heart when administered in combination with GLP‐1 agonism has remained uninvestigated to date. Therefore, we set out to measure the effect of glucagon on postischaemic recovery of contractile function, with and without concomitant GLP‐1 receptor agonism, in the rat heart, which has been demonstrated to respond inotropically to glucagon, and which has a more positive force–frequency correlation with the human heart than the mouse heart (Milani‐Nejad & Janssen, 2014; Rodgers et al., 1981).

We hypothesized (a) that glucagon administration would enhance the functional recovery of the Langendorff‐perfused rat heart following ischaemia/reperfusion, and (b) that coadministration with glucagon would augment any enhancement of functional recovery elicited by GLP‐1 receptor agonism.

## 2 METHODS

### 2.1 Animal studies ethical approval

All experiments conformed with the UK Home Office guidelines under the Animals in Scientific Procedures Act and were approved by the University of Cambridge Animal Welfare and Ethical Review Committee.

### 2.2 Materials and reagents

All reagents were obtained from Sigma Aldrich unless otherwise stated.

### 2.3 Heart perfusion

Male Wistar rats (range, 300–350 g) were obtained from a commercial breeder (Charles River, Margate, UK), and housed in conventional cages with a normal 12‐h/12‐h light/dark photoperiod and access to normal rodent chow and water ad libitum. Rats were euthanized by rising CO_2_ levels, with death confirmed by cervical dislocation. Hearts were excised and perfused in the Langendorff mode with Krebs–Henseleit buffer (118 mM NaCl, 4.7 mM KCl, 1.2 mM MgSO_4_, 11 mM glucose, 1.3 mM CaCl_2_, 0.5 mM EDTA, 25 mM NaHCO_3_, 1.2 mM KH_2_PO_4_; pH 7.4) as previously described (Lindsay et al., 2021), and continually gassed with 95% O_2_/5% CO_2_. Hearts were perfused with 250 mL recirculating buffer under a constant pressure of 100 mmHg. Temperature was maintained at 38°C, the core body temperature for a rat (Lomax, 1966), throughout the protocol. Functional parameters were measured using a PVC balloon inserted into the left ventricle. Rate‐pressure product (RPP) was calculated as the left ventricular developed pressure (LVDP) × heart rate.

The ex vivo ischaemia/reperfusion protocol involved 32 min of aerobic perfusion at 100 mmHg, followed by 32 min of 0.3 mL.min^─1^gww^─1^ low‐flow ischaemia, followed by 32 min of aerobic reperfusion at 100 mmHg to assess functional recovery. Compounds (vehicle control, 40 nM glucagon, and 5 nM exenatide, alone or in combination with 40 nM or 200 nM glucagon, each *n* = 3 biological replicates) were administered directly into the recirculating perfusate 12 min before induction of ischaemia. The compounds remained in the buffer for the duration of the experiment. 40 nM was chosen as the glucagon concentration for investigation owing to its therapeutic effects demonstrated in hepatocytes (Boland et al., 2020), and its maximal effect on contractility in the working rat heart (Rodgers et al., 1981). 200 nM glucagon was used in combination with exenatide as antagonistic signaling pathways may have necessitated a higher concentration when agonists were administered in combination. The concentration of exenatide was 5 times higher than its half maximal effective concentration at the GLP‐1 receptor, and 16.6 times higher than a concentration previously shown to be effective in the Langendorff preparation (Darwesh et al., 2018).

To investigate whether glucagon‐mediated inotropism persisted long enough to explain any improvement in functional recovery, a separate set of hearts (*n* = 6) were perfused for 64 min in aerobic conditions, with glucagon (40 nM) being administered via the perfusion buffer at 30 min.

All results are expressed as mean [SD] (SD = standard deviation). Variance testing indicated unequal variance, therefore statistics were determined using the Welch's one‐way ANOVA and post‐hoc comparisons to test our hypotheses.

## 3 RESULTS

### 3.1 Effect of glucagon upon contractile function and recovery

All hearts displayed consistent absolute cardiac function during the 5 min before the addition of compound or vehicle, with no difference between groups by the Welch's one‐way ANOVA. Mean LVDP [SD] was 127.6 [22], 107.9 [14], 138.6 [4], 123.7 [10], and 129.8 [13] mmHg for groups administered vehicle, 40 nM glucagon, 5 nM exenatide, 5 nM exenatide plus 40 nM glucagon and 5 nM exenatide plus 200 nM glucagon, respectively. In the same group order, the mean heart rate was 280.5 [33], 294.1 [36], 292.7 [43], 308.5 [50], and 285.5 [19] bpm, respectively, while the mean RPP was 35,700 [10000], 33,900 [9000], 38,600 [4000], 38,700 [2000], and 34,900 [4000] mmHg.bpm, respectively.

**FIGURE 1:**
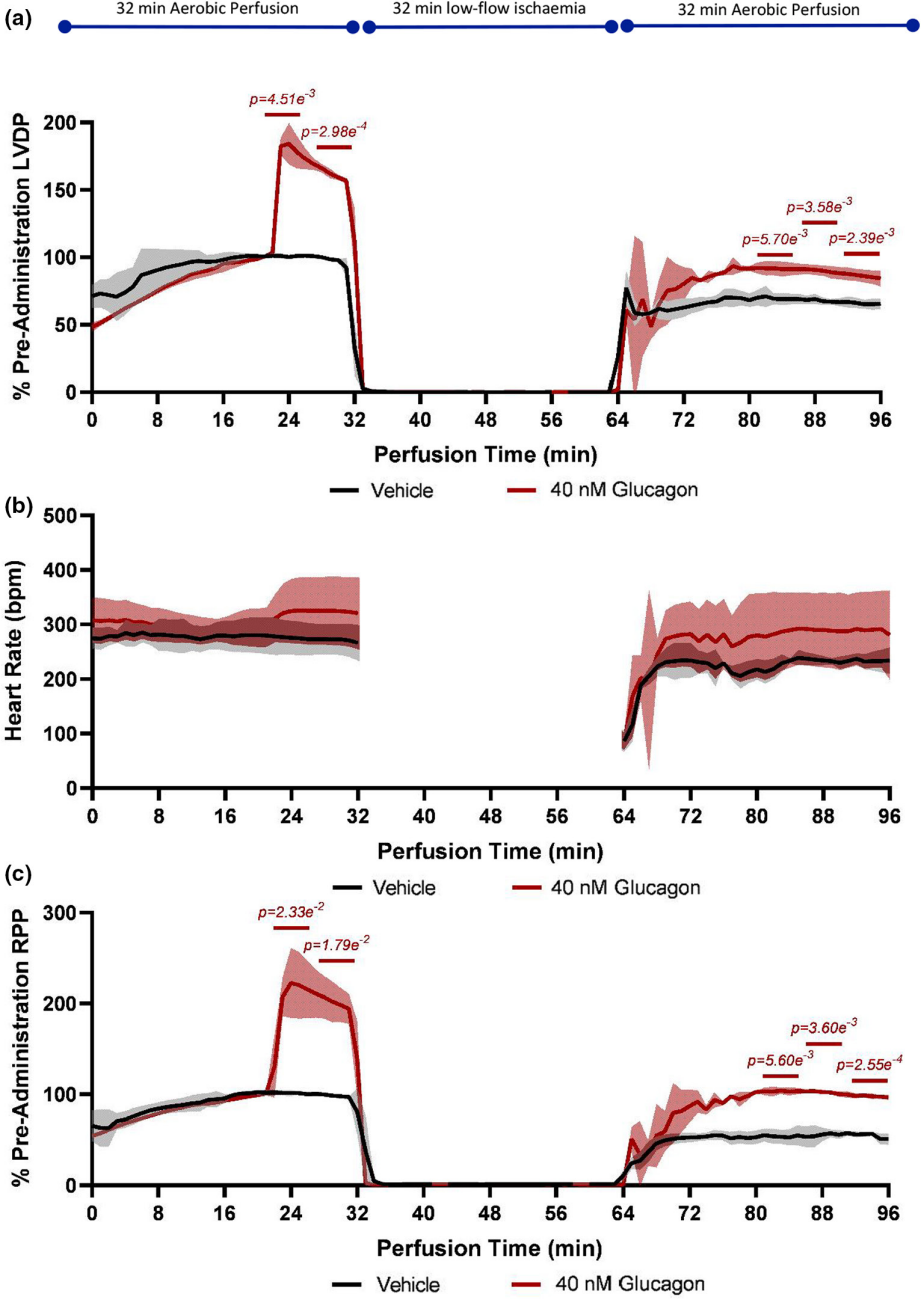
Effects of glucagon upon cardiac contraction and contractile recovery from ischaemia/reperfusion. (a) Percentage of pre‐administration left ventricular developed pressure, (b) heart rate (bpm), and (c) percentage of pre‐administration rate‐pressure product during aerobic perfusion and following ischaemia/reperfusion. At 12 min before the induction of ischaemia, hearts were administered either 40 nM glucagon or vehicle control. RPP was calculated as LVDP multiplied by heart rate. Significance, as determined by the Welch's ANOVA and post‐hoc Welch's correction, is denoted on the graph for the three 5‐minute periods at the end of reperfusion, and the 10 min prior to induction of ischaemia. Data are mean ± SD; *n* = 3 hearts per group. LVDP, left ventricular developed pressure; RPP, rate–pressure product; SD, standard deviation.

Administration of 40 nM glucagon during aerobic perfusion produced two notable effects. First, there was an acute inotropic effect on the heart, with a mean 84%, 38%, and 120% increase in LVDP, heart rate, and RPP, respectively, compared with control (LVDP, *p* = 2.98 e^−4^; heart rate, ns; RPP, *p* = 1.79 e^−2^; Figures 1a–c). This enhancement was evident throughout the 12 min preceding the induction of ischaemia. Second, following ischaemia/reperfusion, there was a 33%, 49%, and 66% improvement in the recovery of LVDP, heart rate, and RPP, respectively, in hearts administered with 40 nM glucagon compared with vehicle (LVDP, *p* = 2.39 e^−3^; heart rate, ns; and RPP, *p* = 2.55 e^−4^, respectively; Figures 1a–c). This represented >90% and 100% recovery of preischaemic LVDP and RPP, respectively, compared with a 50% recovery in control hearts.

### 3.2 Interaction between glucagon and exenatide

**FIGURE 2:**
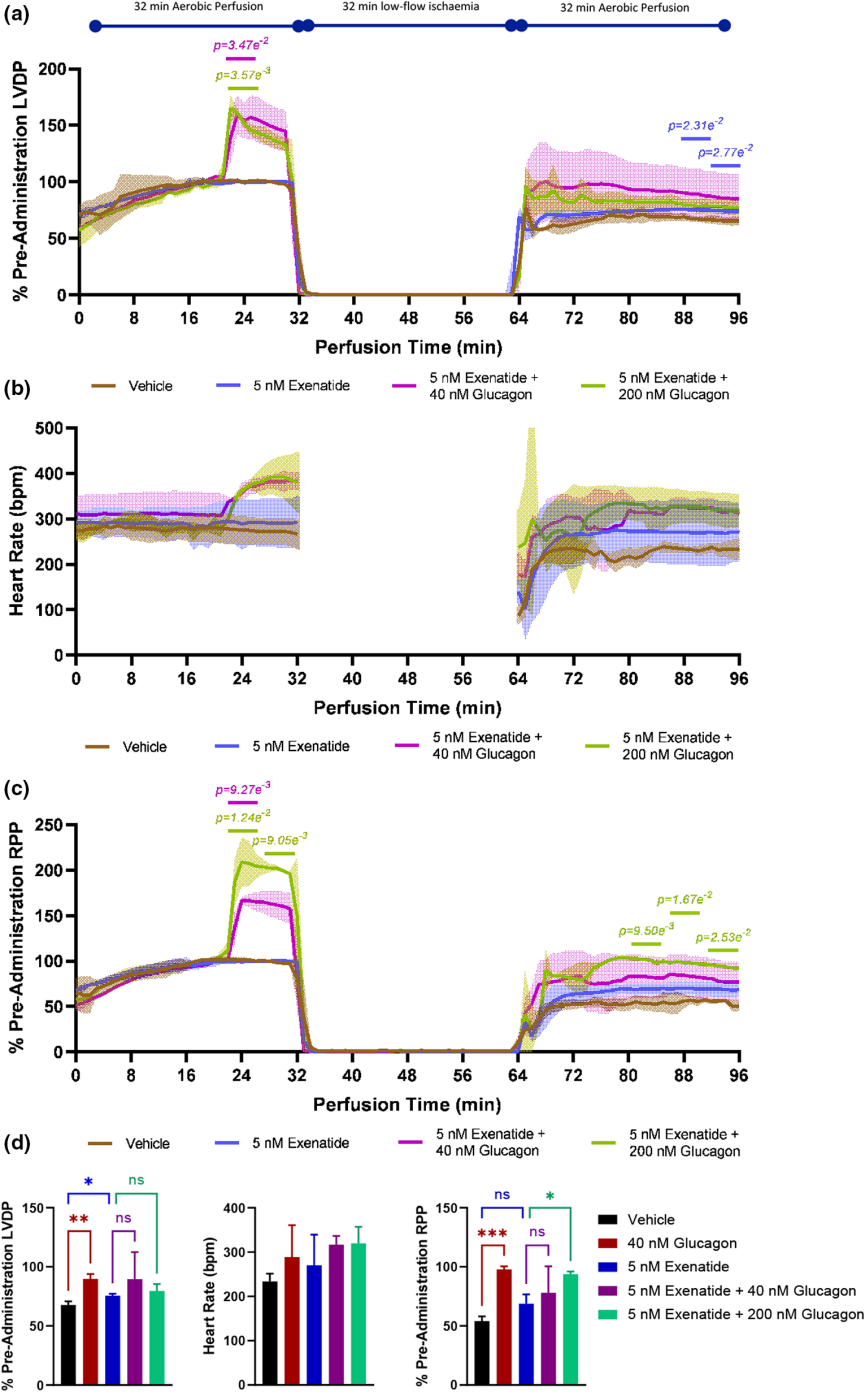
Contractile effects of coadministration of glucagon with exenatide. (a) Percentage of pre‐administration of LVDP, (b) heart rate (bpm), and (c) percentage of pre‐administration RPP during aerobic perfusion and following ischaemia/reperfusion. At 12 min before induction of ischaemia, hearts were administered either 5 nM exenatide alone or in combination with 40 nM or 200 nM glucagon or vehicle control. RPP was calculated as LVDP multiplied by heart rate. Significance, as determined by the Welch's ANOVA and post‐hoc Welch's correction, is denoted on the graph for the three 5‐min periods at the end of reperfusion, and the 10 min prior to induction of ischaemia. Blue represents post‐hoc significance of 5 nM exenatide relative to vehicle; green represents post‐hoc significance of 200 nM glucagon plus 5 nM exenatide relative to 5 nM exenatide. (d) Represents the mean recovery of LVDP, heart rate, and RPP averaged across the 5‐min period before 96 min perfusion time. *p < 0.05, **p < 0.01, and ***p < 0.001. Red represents post‐hoc significance of 40 nM glucagon relative to vehicle, blue represents post‐hoc significance of 5 nM exenatide relative to vehicle, and green represents post‐hoc significance of 200 nM glucagon plus 5 nM exenatide relative to 5 nM exenatide. All data are mean ± SD; *n* = 3 hearts per group. bpm, beats per minute; LVDP, left ventricular developed pressure; RPP, rate–pressure product; SD, standard deviation.

The administration of 5 nM exenatide alone had no measurable inotropic or chronotropic effect in the isolated perfused rat heart (Figure 2a–c). However, following ischaemia/reperfusion, 5 nM exenatide mildly enhanced recovery of contractile function at 96 min, with 16% greater recovery of LVDP relative to vehicle (*p* = 2.77 e^−2^; Figure 2a). While 40 nM glucagon co‐administered with 5 nM exenatide did not further enhance the contractile recovery relative to 5 nM exenatide alone, the administration of 200 nM glucagon alongside 5 nM exenatide instigated 34% greater RPP recovery relative to the addition of 5 nM exenatide alone (Figure 2c, *p* = 2.53 e^−2^).

### 3.3 Contribution of Inotropism to improved functional recovery

**FIGURE 3:**
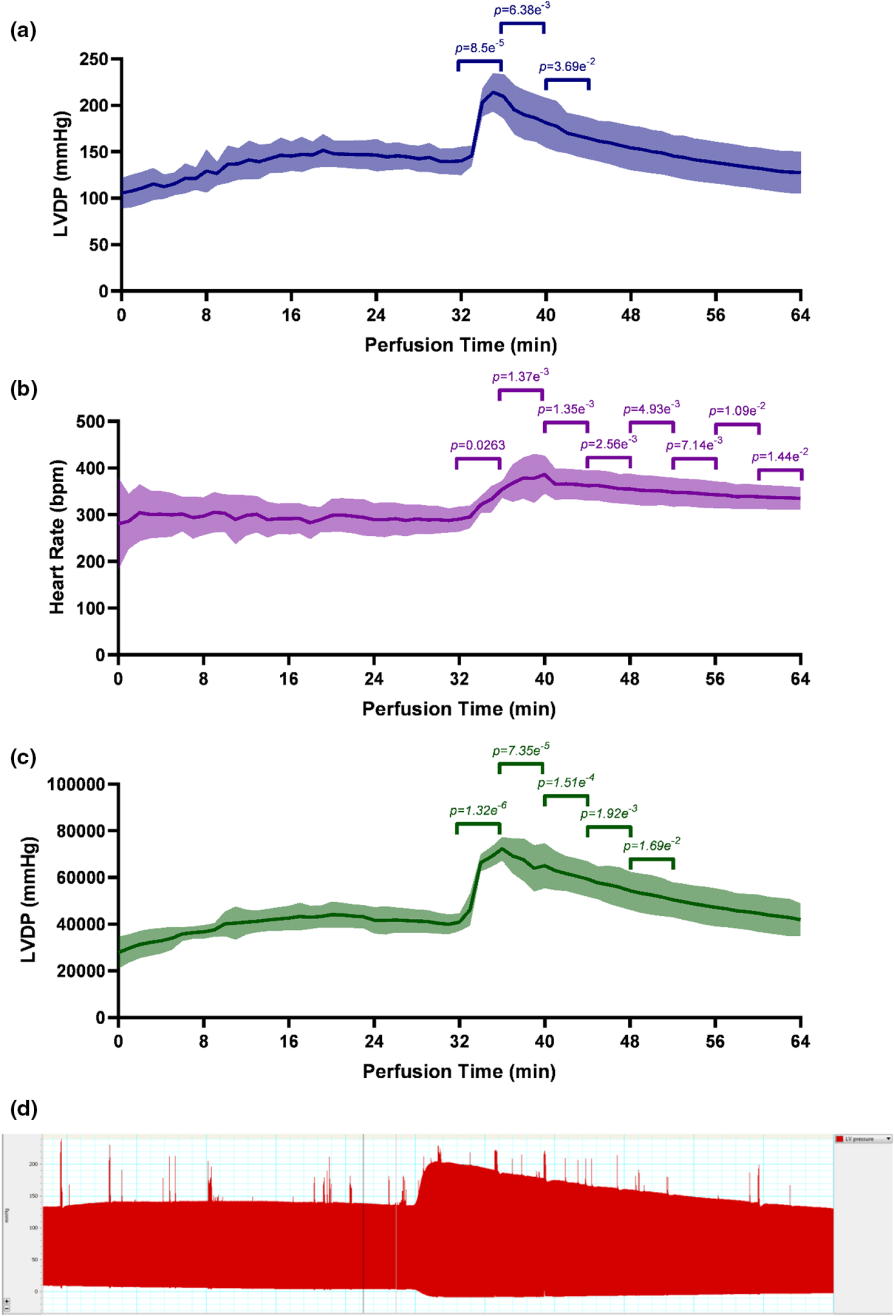
Duration of the contractile effects of glucagon. (a) LVDP (mmHg), (b) heart rate (bpm), and (c) RPP (mmHg.bpm) over 30 min aerobic perfusion without glucagon, and 34 min aerobic perfusion with 40 nM glucagon in the perfusion buffer. (d) Example trace showing left ventricular pressure (mmHg) before and after 40 nM glucagon administration. RPP was calculated as LVDP multiplied by heart rate. Exact statistical significance is annotated on the graph as relative to the 5‐min period before glucagon administration, as determined by the Student's *t*‐test for each subsequent 5‐min period following compound administration. Data are represented as mean ± SD; *n* = 6 hearts. bpm, beats per minute; LVDP, left ventricular developed pressure; RPP, rate–pressure product; SD, standard deviation.

To determine whether the improved functional recovery following ischaemia/reperfusion (sections 3.1 and 3.2) might be explained by the inotropic effect of glucagon persisting postreperfusion, hearts were perfused aerobically for 64 min with glucagon (40 nM) administered at 30 min perfusion time (Figures 3a–c). The inotropic effect of glucagon persisted for <20 min, with both LVDP and RPP returning to pre‐administration levels within 12 and 20 min, respectively (Figures 3a,c). The chronotropic effect of glucagon also declined over time, though heart rate remained 16.1% higher than in control hearts following 34 min (Figure 3b, *p* = 1.44 e^−2^).

## 4 DISCUSSION

These results show that glucagon elicited a strong inotropic response in the rat heart, both alone and when coadministrated with the GLP‐1 receptor agonist exenatide. Glucagon improved postischaemic recovery of cardiac contractility, and also enhanced the modest increase in contractile recovery mediated by GLP‐1 receptor agonism when co‐administered. This improvement of postischaemia/reperfusion function could not be accounted for by inotropic effects of glucagon persisting after ischaemia/reperfusion. In aerobically perfused hearts, the inotropic effect of glucagon ceased after 20 min, while hearts recovering from ischaemia/reperfusion still exhibited improved contractile recovery 76 min after glucagon administration.

We cannot definitively exclude off‐target effects of glucagon such as cross‐talk with the GLP‐1 receptor as detailed by Selley et al. (2016). However, agonism of the GLP‐1 receptor alone by exenatide did not result in any inotropism, which suggests that the effect of glucagon is not mediated via the GLP‐1 receptor. Furthermore, our study used a lower concentration of glucagon than others (Ali et al., 2015), which could be reasonably expected to limit any potential off‐target effects. GLP‐1 antagonists, or, ideally, different unimolecular agonists with known ratios of glucagon:GLP‐1 receptor agonism, could confirm this observation in the future.

Our study, while limited in scope, provides important context for the field. We add to early observations of the positive inotropic effects of glucagon in dogs and rats (Farah & Tuttle, 1960; Rodgers et al., 1981). Our results also fit alongside reports of faster restoration of vasodilation postischaemia/reperfusion in isolated rat hearts administered 400 nM glucagon (Rosic et al., 2010). On the other hand, our findings contrast those of a previous study in the perfused mouse heart, which suggested that exogenous glucagon may impair contractile recovery, while glucagon receptor deletion may be protective (Ali et al., 2015). The same report observed no inotropic effect despite the use of a five‐fold higher concentration of glucagon. The differing inotropic responses from the hearts of the two rodent species may act as an indicator for how susceptible they are to glucagon‐mediated improvement of cardiac recovery post‐I/R. Rat hearts have a more positive force–frequency relationship with humans than mice do, and are better able to increase heart contractility in response to exercise (Milani‐Nejad & Janssen, 2014), which may make them better able to respond to glucagon. Further, it is possible that the improved survival reported in mice with cardiac‐specific deletion of the glucagon receptor may relate more to differing heart development than to loss of acute glucagon signaling at the time of the ex vivo ischaemic incident (Ali et al., 2015).

Studies in humans have been similarly contradictory, with some demonstrating a lack of response to glucagon and others demonstrating inotropism alongside cardioprotection (Lvoff & Wilcken, 1972; Parmley et al., 1968). Goldstein et al. (1971) demonstrated that the responsiveness of human heart tissue to glucagon declined as heart failure progressed, which may explain some of these inconsistencies, especially since the studies reporting little response were carried out in patients with low cardiac output. In the multiple ongoing clinical trials of therapeutics with glucagon agonist function, there has been no increase in adverse cardiac events, making it unlikely that glucagon has a detrimental influence on the human heart (Parker et al., 2020, 2022).

GLP‐1, glucagon, and GIP combinations are well‐established pharmacologically and exhibit effects, which could be pertinent for diseased hearts. Since the inotropic effect of glucagon does not fully account for the preserved contractile function we observed postischaemia/reperfusion, it may be that differences in their physiological signaling mechanisms underlie any synergy. GLP‐1 agonism decreased cAMP in the heart (Aravindhan et al., 2015), while glucagon has been shown to boost cardiac cAMP levels (Farah & Tuttle, 1960), so this may counteract a possible downside of GLP‐1 signaling. In the alternative scenario of the diabetic heart, where glucose metabolism is dysregulated, enhancement of glycolysis while preserving cAMP function may further help protect against ischaemia/reperfusion injury. Glucagon signaling, alone and in concert with GLP‐1 agonism, has also been shown to enhance autophagy and rejuvenation of dysfunctional mitochondrial populations (Boland et al., 2020), so it is possible that this aids recovery from ischaemia/reperfusion via removal of damaged, ROS producing organelles. It would be worthwhile to look at metabolite usage, markers of cell death, and cAMP signaling in future studies.

In summary, these results show that glucagon administration mediates an improvement of contractile recovery following ischaemia/reperfusion in the isolated perfused rat heart. Glucagon also enhanced the mild improvement of contractile recovery attained with a GLP‐1 receptor agonist. Notable limitations of our study are the use of a single methodology and the absence of in vivo experiments. However, our study contributes to the overall picture surrounding the influence of glucagon and GLP‐1 agonism upon the heart. To explain similarities and discrepancies between previous studies, the field requires a rigorous comparison of glucagon's cardiac effects between species, and between different heart conditions (failing heart vs. healthy or diabetic).

## AUTHOR CONTRIBUTIONS

RTL and AJM contributed to experimental conception and design and funding acquisition. RTL contributed to the acquisition, analysis, validation, and interpretation of data and drafting of the article. All authors contributed to the critical review of the article and approved the final version for submission.

## FUNDING INFORMATION

This work was supported by British Heart Foundation (FS/14/59/31282), Research Councils UK (EP/E500552/1), and MedImmune.

## CONFLICT OF INTEREST STATEMENT

RTL is affiliated with BioPharmaceuticals R&D, AstraZeneca, and declares no other competing financial interests. PA and LJ are employees and shareholders of AstraZeneca. MedImmune provided funding for this research and had no role in study design, data collection, analysis, and interpretation. AJM received support from MedImmune but declares no other competing interests.
